# Association of human papillomavirus and *Chlamydia trachomatis* with intraepithelial alterations in cervix samples

**DOI:** 10.1590/0074-02760150330

**Published:** 2016-02

**Authors:** Denise Wohlmeister, Débora Renz Barreto Vianna, Virgínia Etges Helfer, Fabrícia Gimenes, Marcia Edilaine Lopes Consolaro, Regina Bones Barcellos, Maria Lucia Rossetti, Luciane Noal Calil, Andréia Buffon, Diogo André Pilger

**Affiliations:** 1 Universidade Federal do Rio Grande do Sul, Programa de Pós-Graduação em Ciências Farmacêuticas, Porto Alegre, RS, Brasil; 2 Universidade Federal do Rio Grande do Sul, Faculdade de Farmácia, Departamento de Análises, Laboratório de Análises Bioquímicas e Citológicas, Porto Alegre, RS, Brasil; 3 Universidade Federal do Rio Grande do Sul,Faculdade de Farmácia, Porto Alegre, RS, Brasil; 4 Universidade Estadual de Maringá, Laboratório de Citologia Clínica, Maringá, PR, Brasil; 5 Fundação Estadual de Produção e Pesquisa em Saúde, Centro de Desenvolvimento Científico e Tecnológico, Porto Alegre, RS, Brasil

**Keywords:** HPV, C. trachomatis, intraepithelial lesions, sexually transmitted diseases

## Abstract

The influence of different infectious agents and their association with human papillomavirus (HPV) in cervical carcinogenesis have not been completely elucidated. This study describes the association between cytological changes in cervical epithelium and the detection of the most relevant aetiological agents of sexually transmitted diseases. Samples collected from 169 patients were evaluated by conventional cytology followed by molecular analysis to detect HPV DNA, *Chlamydia trachomatis*, herpes simplex virus 1 and 2,*Neisseria gonorrhoeae*, *Mycoplasma genitalium*, *Trichomonas vaginalis*, and*Treponema pallidum*, besides genotyping for most common high-risk HPV. An association between cytological lesions and different behavioural habits such as smoking and sedentariness was observed. Intraepithelial lesions were also associated with HPV and *C. trachomatis* detection. An association was also found between both simple and multiple genotype infection and cytological changes. The investigation of HPV and *C. trachomatis*proved its importance and may be considered in the future for including in screening programs, since these factors are linked to the early diagnosis of patients with precursor lesions of cervical cancer.

Cervical cancer is the third most common neoplasia worldwide. Specifically in Brazil, the disease ranks third, and in southern region of the country it is the fifth most prevalent, with 16 cases for every 100,000 women. Early detection of epithelial lesions in the cervix has proved its value, since patient benefit is strongly correlated with intraepithelial lesions grade as defined upon diagnosis (Bringhenti et al. 2010, INCA 2014). Several factors affect cervical carcinogenesis, from behavioural variables to the presence of infectious agents linked with sexually transmitted diseases (STDs). This is true especially for high carcinogenesis risk genotypes of the human papillomavirus (HPV) (Al-Daraji & Smith 2009, Schiffman & Solomon 2013). The specialised literature reports ample evidence that the incidence of HPV is higher in women with secondary genital infections. The vaginal microenvironment may be considered a cofactor in the pathogenesis of cervical intraepithelial neoplasia (Murta et al. 2001, Martins et al. 2007, Campos et al. 2008 *).* The influence of different infectious agents and their association with HPV in cervical carcinogenesis has not yet been fully explained, and rare are the studies that evaluate the potential applicability the detection of such agents as a tool in screening programs.

It is believed that persistent HPV infection in the cervical epithelium is facilitated by inflammatory processes caused by other STD pathogens. Among the main aetiological agents responsible for STDs that may be potentially involved in cervical carcinogenesis are*Chlamydia trachomatis*, herpes simplex virus (HSV) 1 and 2,*Neisseria gonorrhoeae*, *Mycoplasma genitalium*,*Trichomonas vaginalis*, and *Treponema pallidum*, which cause inflammatory processes and microabrasion or microtrauma on the cervical epithelium, deteriorating the infection scenario and promoting the persistence of HPV (Muvunyi et al. 2011, Rodríguez-Cerdeira et al. 2012).

Cervical Pap smears afford to visualise the presence of microorganisms or the cell changes they cause, though the technique sometimes is not sensitive enough to determine the actual aetiological agent of these diseases (Martins et al. 2007). When used as a complementing tool to conventional cytology, new methods, especially molecular techniques, show good promise in more unambiguous identification of microorganisms with a likely role in cervical carcinogenesis (Rodrigues et al. 2009).

In this scenario, the present study aimed to investigate the relationship between degree of cytological changes in the cervical epithelium and the presence of the most important aetiological pathogens of STDs.

## SUBJECTS, MATERIALS AND METHODS

*Samples* - A cross-sectional study among women from one private medical unit in the municipality of Carazinho, southern Brazil. A total of 169 ectocervical and endocervical samples were collected during routine gynaecologic examination from 169 patients between March-November 2014. Participants included women at reproductive age who agreed to participate voluntarily after signing an informed consent form.

After answering a questionnaire, patients were submitted to the routine collection of cervical specimens. These samples were mounted on cytology slides in a liquid medium kit (DigeneDNA with PAP^®^ - DNA Collection Device - HC2 HPV and CT/GC DNA Tests; QUIAGEN, EUA) and used for molecular analyses. Exclusion criteria were different age from the age groups defined in the study, the refusal to participate, and pregnancy due to factors that may interfere in the results of analyses.

*Conventional cytology* - Cervical Pap smears were mounted on slides and stained using the Papanicolaou stain as described by Consolaro and Engler (2012). Optical microscopy was used to evaluate cellular changes, which were interpreted and classified according to the 2001 Bethesda System (Solomon & Nayar 2005). Slides were inspected by double-blinded independent cytologists. The interobserver agreement index was assessed based on the classification of ectocervical samples as “negative for intraepithelial lesion or malignancy” [“within normal limits” (WNL) or “reactive or inflammatory benign cellular changes” (RI)], “atypical squamous cells of undetermined significance” (ASC-US), “low-grade squamous intraepithelial lesions” (LSILs), “cannot exclude high-grade squamous intraepithelial lesion” (ASC-H), “high-grade squamous intraepithelial lesion” (HSIL), and “squamous cervical carcinoma” (CC). When detected, endocervical changes were classified into “atypical glandular cells of undetermined significance” (AGUS), “adenocarcinoma in situ”, or “invasive adenocarcinoma”.

*Molecular analysis* - *DNA extraction* - Samples of the endocervical and ectocervical regions were placed in specific liquid medium and refrigerated at 4-8ºC until analysis. DNA was extracted using the commercial kit Qiamp DNA Mini Kit (QIAGEN, Germany) according to the manufacturer’s instructions.

As internal control and a means to ensure quality in the DNA extraction process the β-actin housekeeping gene was amplified for all samples as described by Brugè et al. (2011).

*Detection of HPV DNA* - The qualitative screening test to detect HPV DNA was carried out by conventional polymerase chain reaction (PCR) adapted fromShen-Gunther and Yu (2011). The reaction amplifies the *L1* gene to simultaneously detect low and high-risk HPV DNA, though it does not differentiate the various viral types present in the sample. Briefly, the PCR was carried out using the primers MY09 (5′-CGTCCMARRGGAWACTGATC-3′) (forward) and MY11 (5′-GCMCAGGGWCATAAYAATGG-3′) (reverse) to a final 25 μL volume under the following amplification conditions: preheating (1 min at 95ºC), 35 cycles (1 min at 94ºC, 1 min at 60ºC, 1 min at 72ºC), and final extension (10 min at 72ºC). Finally, 450 bp fragments were observed on 2% agarose gels under ultraviolet light.

*HPV genotyping* - The samples that tested positive for HPV in the PCR were investigated for the presence of the most common high oncogenic risk HPV genotypes (HPV 16, 18, 31, 33, 39, and 45) using the microplate colorimetric hybridisation assay (MCHA) as described by Barcellos et al. (2011). First, a 150 bp fragment of the HPV L1 region was amplified using the consensus biotynilated GP5+ and GP6+ primers, followed by hybridisation on microplate containing specific probes for subsequent detection by colorimetry. Amplified fragments were individually hybridised using a probe specific to each viral type in the microplate and, after incubation and rinsing steps, absorbance was read at 450 nm (secondary absorbance at 620 nm) in an automatic microplate reader. The cut-off value for each probe was considered in the analysis and interpretation of respective results and the positive and negative controls used were described in the standardisation of the technique by Barcellos et al. (2011).

*Detection of other STD pathogens* - The DNA extracted from cervical samples was also used to detect other infectious agents potentially associated with HPV. We designed a multiplex-PCR assay to achieve simultaneous detection of the seven selected STDs: *N. gonorrhoeae*, *T. pallidum*,*C. trachomatis*, *T. vaginalis*, *M. genitalium*, HSV1, and HSV2 previously standardised and validated by our team members. The sizes of the expected fragments and the sequences of primers used were described and standardised in previous studies (Souza et al. 2013, Gimenes et al. 2014).

*Statistical analyses* - Data were analysed using the Statistical Package for Social Sciences software v.18.0. Qualitative variables were described as means and standard deviation, and compared using Fischer’s exact test. The association between variables was evaluated using Pearson’s correlation coefficient. Confidence interval was set at 95% (p < 0.05). Interobserver agreement used in the cytological diagnosis was calculated by confidence statistics based on Cohen’s kappa (k), with k > 0.8 meaning “excellent”, k = 0.6-0.8 meaning “good”, k = 0.4-0.6 meaning “average”, and k < 0.4 meaning “poor”.

*Ethics* - This research was approved by the Ethical Committee of the Federal University of Rio Grande do Sul, Brazil (protocol 562.824) and carried out according to the 1975 Declaration of Helsinki, 2000 revision.

## RESULTS

The patients mean age was 33 ± 11 years, varying between 15-64 years. Socioeconomic, behavioural, and clinical profiles of patients are shown in Table I.

**TABLE I:** Sociodemographic characteristics, behavioural, and clinical of patients

	Frequency n (%)
Age group (years)	
15-20	16 (9.5)
21-30	60 (35.5)
31-40	43 (25.4)
41-50	29 (17.2)
>50	16 (9.5)
NI	5 (3)
Level of education	
Elementary school	43 (25.4)
High school	56 (33.1)
Higher education	68 (40.2)
NI	2 (1.2)
Marital status	
Single	47 (27.8)
Married	92 (54.4)
Steady partner (1 year)	16 (9.5)
Divorced	10 (5.9)
Widow	4 (2.4)
Smoking	
Yes	18 (10.6)
No	149 (88.2)
NI	2 (1.2)
Regular physical activity (2-3 times/week)	
Yes	64 (37.9)
No	102 (60.4)
NI	2 (1.8)
Age of earlier sexual activity (years)	
10-14	20 (11.8)
15-20	125 (74)
21-30	18 (10.6)
NI	6 (3.5)
Sexual partners during life	
1-2	84 (49.7)
3-5	44 (26)
6-10 p	15 (8.9)
11-15	1 (0.6)
> 16	4 (2.4)
NI	21 (12.4)
Offspring	
No	66 (39)
1-2	82 (48.5)
3-5	18 (10.6)
6-10	2 (1.2)
NI	1 (0.6)
Contraceptive method	
None	38 (22.5)
Oral contraceptive	99 (58.6)
Intra uterine device	5 (3)
Condom	8 (4.7)
Sterilisation	8 (4.7)
Injectable	11 (6.5)
Hormone replacement	
Yes	9 (5.3)
No	158 (93.5)
NI	2 (1.2)
HPV infection history	
Yes	35 (20.7)
No	131 (77.5)
NI	3 (1.8)
Uterus cancer history	
Yes	1 (0.6)
No	166 (98.2)
NI	2 (1.2)
Uterine cancer in family	
Yes	28 (16.6)
No	136 (80.5)
NI	5 (3)
Symptoms - vaginal discharge/itching	
Yes	56 (33.1)
No	104 (61.5)
NI	9 (5.3)

HPV: human papillomavirus; NI: not informed.

**Fig. 1:**
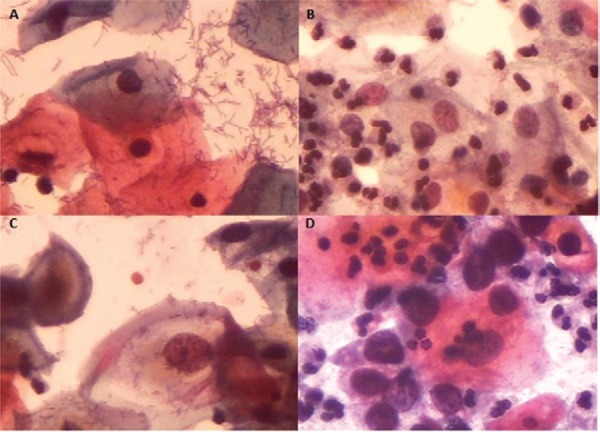
: cytological samples stained using the Papanicolaou technique and classified into three groups according to the 2001 Bethesda System (400X): A: negative for intraepithelial lesion or malignancy within normal limits; B: negative for intraepithelial lesion or malignancy with reactive or inflammatory benign cellular changes; C: low-grade squamous intraepithelial lesion; D: high-grade squamous intraepithelial lesion.

*Conventional cytology* - The cytological diagnosis was conducted by three independent cytologists who shared excellent interobserver agreement (k = 0.860). Of the 169 patients, 151 (89.3%) presented negative results for intraepithelial lesions or malignancy, of which 75 (49.7%) were WNL and 76 (50.3%) had RI. Eighteen of the 169 samples analysed (10.6%) were categorised as cytological alterations/changes, which in turn were divided into four (22.2%) ASC-US, 10 (55.5%) LSIL, one (5.5%) ASC-H, and three (16.6%) HSIL. The results were sorted into three distinct groups: (A) WNL, (B) RI, and (C) atypia or lesions (which included ASC-US, LSIL, ASC-H, and HSL), as shown in Fig. 1.

Epidemiological variables were compared to the cytological diagnosis when some relationships became apparent. The results show that group A was formed mostly by patients with higher education degrees (p < 0.05). Patient sedentariness and reported vaginal discharges and pruritus were prevalent in group B (p = 0.036 and 0.055, respectively). In group C, more specifically the patients with positive ASC-US diagnosis, most individuals were smokers (p < 0.05), as shown in Table II.

**TABLE II:** Cytological diagnosis and epidemiological variables

Cytological diagnosis group per group	Samples n (%)	Epidemiological variable [n (%)]	p
A	75 (44.4)	Higher education degrees [18 (24)]	< 0.05
B	76 (45)	Sedentariness [49 (64.5)] Vaginal discharges/pruritus [38 (50)]	0.036 0.055
C	18 (10.6)	Smokers [14 (77.7)]	0.041

group A: within normal limits; group B: reactive or inflammatory benign cellular changes; group C: atypia or lesions (which included atypical squamous cells of undetermined significance, low-grade squamous intraepithelial lesions, cannot exclude high-grade squamous intraepithelial lesion, and high-grade squamous intraepithelial lesion); HPV: human papillomavirus.

*Molecular analysis* - *HPV detection* - All samples analysed were positive for the β-actin housekeeping gene and were analysed to detect HPV DNA. Of the 169 samples, 35 (20.7%) presented high and/or low oncogenic risk HPV DNA, present either alone or in conjunction with other viral types. In addition, the comparison with the cytological diagnosis demonstrated an association between HPV DNA detection and group C, which comprised the different classes of atypia and cytological lesions (ASC-US, LSIL, ASC-H, and HSIL) (p < 0.001), as shown inTable III. The HPV DNA detection frequency was 28.6% in cases of LSIL. The age group with the highest frequency of cytological changes and HPV detection was the 21-30-year-old group, although no statistically significant relationship was observed. Some samples with negative cytology also were positive in HPV DNA detection (11.2%).

**TABLE III:** Cytological diagnosis and human papillomavirus (HPV) infection

HPV distribution		Group A (n = 75) n (%)	Group B (n = 76) n (%)	Group C (n = 18) n (%)
HPV DNA prevalence	35 (20.7)	9 (11.7)	8 (10.8)	18 (100)
HPV16	21 (60)	5 (6.5)	4 (5.4)	12 (66.7)
HPV18	(25.7)	0 (0)	2 (2.7)	7 (38.9)
HPV31	11 (31.4)	1 (1.3)	3 (4.1)	7 (38.9)
HPV33	9 (5.3)	1 (1.3)	3 (4.1)	5 (27.8)
HPV39	22 (62.8)	6 (7.8)	8 (10.8)	8 (44.4)
HPV45	3 (8.6)	1 (1.3)	0 (0)	2 (11.1)
Multiple HPV infection	15 (42.8)	2 (2.6)	3 (4.1)	10 (55.6)

group A: within normal limits; group B: reactive or inflammatory benign cellular changes; group C: atypia or lesions (which included atypical squamous cells of undetermined significance, low-grade squamous intraepithelial lesions, cannot exclude high-grade squamous intraepithelial lesion, and high-grade squamous intraepithelial lesion); HPV: human papillomavirus.

Genotype distribution analysis showed that 35 cases of infection were identified as different types of HPV infection as well as simple co-infection. Among these infections, the genotype of HPV 39 was more prevalent, being detected in 22 samples (62.8%), while HPV 16 was detected in 21 (60%), HPV 31 in 11 (31.4%), HPV 18 in nine (25.7%), and HPV 45 in three (8.6%) samples (Table III).

Among the patients positive for HPV DNA, 13 (37.1%) had only one genotype of the virus, while 20 (57.1%) presented more than one viral type. When infection with multiple genotypes is considered, the most frequent associations were HPV 16/39 in seven (20%) samples, HPV 16/18/31/33/39 in four (11.4%), and HPV 16/18/31/33 in three (8.6%) samples.

Of the 35 samples that were diagnosed positive for HPV, two (5.7%) were not positive for the high-risk HPV genotypes investigated. HPV 16 was detected as single genotype in only three (8.6%) of samples. In the other specimens analysed, the genotype occurred together with other HPV types, as multiple infection. Single infection with HPV 18 and HPV 31 was not observed.

The comparison between cytological results and HPV genotype showed that group C presented an association with infection with both single and multiple viral genotypes (p = 0.0001). Also, an association between ASC-US and multiple infection with HPV 31/39 and HPV 16/18/31/33/39 was observed. LSIL was associated with HPV 16, HPV 39, and HPV 45, and with multiple infections with HPV 16/18/31/33 and HPV 16/39/45. ASC-H was associated with multiple infection with HPV 16/18, while HSIL was linked with multiple infection with HPV 16/18/31/33 (p = 0.036).

*Detection of STD pathogens* - As shown on Table IV, after HPV, *C. trachomatis* is the most prevalent aetiological agent STD detected in the samples, either as a single pathogen or in co-infection. *T. vaginalis*, *N. gonorrhoeae*, *M. genitalium*, HSV1, HSV2, and *T. pallidum* were found at lower frequency. The cases of co-infection with*C. trachomatis,* HPV, HSV1, HSV1 and *T. pallidum*, and *M. genitalium* are depicted on Table IV.

**TABLE IV:** Frequency distribution of infectious agents

Multiplex	Cases n (%)
Negative	98 (58)
HPV	35 (20.7)
*Chlamydia trachomatis*	16 (9.5)
*Trichomonas vaginalis*	8 (4.7)
*Neisseria gonorrhoeae*	2 (1.2)
*Mycoplasma genitalium*	3 (1.8)
HSV1	4 (2.4)
HSV2	1 (0.6)
*Treponema pallidum*	2 (1.2)

Total	169 (100)

Co-infections	
*Chlamydia trachomatis +* HPV *Chlamydia trachomatis +* HSV1	4 (2.4) 1 (0.6)
*Chlamydia trachomatis +* HSV1 +*Treponema pallidum*	1 (0.6)
*Chlamydia trachomatis + Mycoplasma genitalium*	1 (0.6)

Total	7 (4.2)

HPV: human papillomavirus; HSV: herpes simplex virus.

**Fig. 2:**
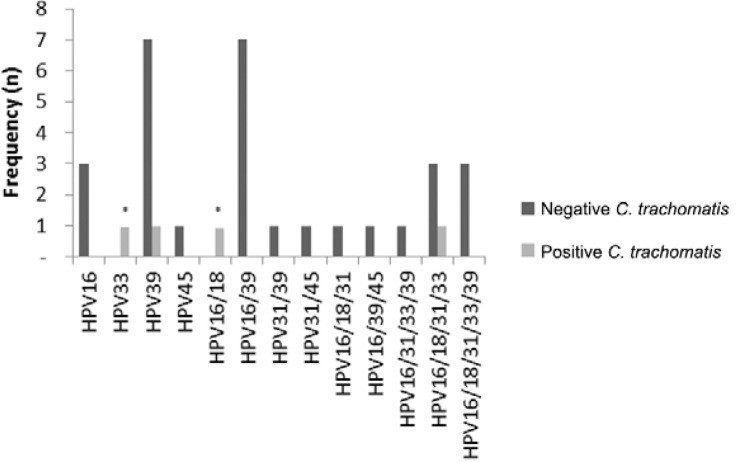
: frequency distribution of *Chlamydia trachomatis* in samples considering the different high oncogenic risk human papillomavirus (HPV) genotypes. Asterisk means association between HPV and positive result for *C. trachomatis*.

The comparison between cytological findings and the analysis of STD pathogens revealed an association between *C. trachomatis* and ASC-H in group C (p = 0.02). When the presence of high-risk HPV genotypes is considered in light of the detection of STD pathogens, *C. trachomatis* was associated with the presence of HPV 33 and multiple infection, with HPV 16/18. Moreover, higher prevalence of either simple or double infection with HPV 16 and HPV 39 was detected, independently of infection with *C. trachomatis* (Fig. 2). The other pathogens studied showed no statistically significant association.

## DISCUSSION

The present study identifies an important association with cervical cytological changes, presence of HPV and *C. trachomatis*. Such relationship reveals that both agents are linked with early cervical carcinogenesis.

The Pap smears analysed were collected from patients that participated in cervical cancer screening programs. Most women (60.9%) were between 21-40 years of age, which represents an appropriate age group, considering the objectives of screening programs and the fact that infections and cytological changes manifest mainly in this demographic (Batista et al. 2014 *)*.

The analysis of behavioural and clinical profiles of patients shows that most were married women with higher education degrees and who took physical exercise, while few were smokers. Although the cytological diagnosis was normal for roughly half the samples, a large number of patients exhibited active and/or reactive lesions associated with sedentariness and smoking habit. These factors are allegedly associated with a greater incidence and prevalence of HPV infection. Moreover, the higher the number of cigarettes smoked daily, the greater the risk of cervical cancer, especially when the smoking habit started early in life (INCA 2014, Kaderli et al. 2014). Another interesting aspect in the screened population is that signs like increased vaginal discharges and symptoms such as pruritus indicate inflammatory process and should prompt the patient to look for medical care, in which case the investigation into the presence of the infectious agents cited should be requested.

Sexual promiscuity is a confirmed risk factor in the spread of HPV infection (Pinheiro et al. 2013), though most women in the present study reported having had only one or two partners at the time this study was carried out. This finding should be interpreted based on the age of patients had the first intercourse (between 15-20 years old). Also, the most commonly adopted pregnancy control method was the contraceptive pill, as published in previous study (Batista et al. 2014). The age group that presented the highest frequency of cytological changes was between 21-30-years-old. This age series is believed to be the time at which the early manifestations of cervical cancer occur, confirming the importance of preventive exams (Murta et al. 2001, Falcão et al. 2014).

The prevalence of cytological changes observed in the present study may be considered high (10.6%) when compared with the findings published in the literature (de Melo et al. 2009), a prevalence of only 1.2% of squamous or glandular epithelial cell changes. Nevertheless, the prevalence of cytological changes seems to vary across geographical regions, which are covered under different approaches to screening programs. In addition, in some Brazilian states, especially in the South and Northeast regions, such initiatives are allegedly far too infrequent (Correa et al. 2012). It should be stressed that the samples analysed in the present study were collected from follow-up patients, who may have been more prone to manifesting the changes diagnosed. Furthermore, the fact that the participants with a record of changes or infections were more motivated to take part in this research, increasing the frequency of changes observed, should not be ruled out.

Conventional cytology was originally developed and is presently used as a routine screening method to detect cervical changes and precursor lesions to cervical cancer (Og et al. 2010). Further, exfoliative cytology does not provide good sensitivity levels and therefore other techniques are employed, mainly molecular methods. Here, qualitative PCR afforded to detect HPV DNA in 20.7% of the samples, a number that presented good correlation with cytological changes (ASC-US, LSIL, ASC-H, and HSIL), with higher frequency of LSIL (28.6%).

Of the patients with negative diagnosis of intraepithelial lesion or malignancy, 11.2% were positive for HPV DNA, of which 6% were within normal levels and 5.2% presented reactive benign inflammatory changes. A screening effort carried out in 2007 demonstrated the prevalence of 10.4% of HPV in samples collected from patients with normal cytological results across the world (de Sanjosé et al. 2007). The same study revealed that in South America this prevalence was as high as 12.3% (de Sanjosé et al. 2007). This finding draws attention to the possibility to detect HPV before it causes any cytological lesion, in which case patients may be more suitably followed up (Entiauspe et al. 2010). Interestingly HPV infection may likewise be asymptomatic and transient, and spontaneous clearance can occur in 80% of cases, when the virus is flushed by the host’s immune system - with no ensuing cell changes. In roughly 20% of women HPV infection may be persistent and subsequently evolving into cervical cancer in up to 10% of cases (de Abreu et al. 2012b).

High oncogenic risk HPV genotypes may infect the epithelium persistently, inducing lesion progression and contributing to carcinogenesis. Research has shown that the most common high-risk HPV genotypes detected in carcinoma cases are HPV 16, 18, 31, 33, 39, and 45 (Muñoz 2000, Kraus et al. 2006, Parkin et al. 2008, Barcellos et al. 2011). HPV 39, which is quite prevalent in Latin American populations, has been detected at high frequencies both in isolation and in co-infection with other genotypes (Muñoz 2000). HPV 39 is one of the genotypes most commonly associated with LSIL and is held accountable for a mere 3% of cervical cancer worldwide (Clifford et al. 2005). It is important to note that the technique used for genotyping has a limitation with regard to the detection of the genotype HPV 39, which has low agreement with the PapiloCheck technique; however, all samples with HPV 39 were analysed and repeated using the technique MCHA.

In a study carried out in southern Brazil, Entiauspe et al. (2010) demonstrated that HPV 16 was the most prevalent genotype, followed by HPV 18. HPV 16 was also highly prevalent in the population examined in the present study. Besides, it is the main genotype detected in squamous carcinomas (Oliveira-Silva et al. 2011). Interestingly, HPV 18 is among the most prevalent genotype worldwide, though in the samples analysed in the present study it was comparatively less common than other genotypes and was never detected as sole genotype in any of the patients examined. The main underlying reason may be the fact that HPV 18 is involved mostly in cases of adenocarcinoma or adenosquamous carcinoma, which represents only 5% of cervical cancers (Nakagawa et al. 2010).

Relevant findings of the present study include the simultaneous presence of more than one HPV genotype in the same sample and the association of these genotypes with the diagnosis of cytological changes. Here, the presence of one genotype is more prevalent in LSIL cases, while higher-grade lesions were characterised by multiple HPV genotypes. This reveals the importance of identifying the HPV type, since a mere positive (or negative) result does not afford such analysis of the HPV genotype present in the sample. Another important aspect is the possibility that the presence of more than one viral type indicates exposure of a patient to a risk factor. Previous studies have shown that infection with multiple HPV genotypes increases the risk of intraepithelial lesions, though the prevalence of these multiple genotype infections did not vary across the different intraepithelial lesion stages (Schimitt et al. 2013).

Only 5.7% of the samples positive for HPV DNA were not positive for the high-risk HPV genotypes screened, proving the effectiveness of the first reaction. These samples may be considered positive for low risk HPV or positive for other high-risk HPV genotypes that are not detected by the probes used.

STD pathogens are also contributing factors for HPV infection and tumour progression. These pathogens cause inflammatory processes and epithelial lesions, worsening the picture concerning virus lodging and persistence (Entiauspe et al. 2010). The detection of aetiological agents of STDs by amplification of nucleic acids is considered more sensitive than conventional microscopy or culture methods (Schimitt et al. 2014). Among the samples diagnosed with RI, 16.2% were positive in the molecular detection of one or more STD aetiological agents. In turn, 83.8% presented unspecific inflammatory characteristics, and the aetiological agent was not detected. The relationship between smears positive for inflammation and the diagnosis of malignancy has been reported, and the study by Og et al. (2010)suggests that these infections may hide premalignant and malignant changes, requiring treatment of the inflammatory process prior to any cytological diagnosis effort.

Most samples in which an aetiological agent of a STD was detected had negative findings for intraepithelial lesion or malignancy (WNL/RI). A distinct result was observed for HPV infection, which was more prevalent in patients with atypia or intraepithelial lesions. In addition, 53.6% of patients who recounted symptoms like vaginal discharge or pruritus also were positive for one or more pathogens.

The prevalence of the different aetiological agents of STDs observed in the present study draws attention to the high prevalence of *C. trachomatis* and to the relationship this microorganism establishes with HPV. It is believed that*C. trachomatis* infection may affect HPV infection and persistence, since the condition allegedly increases the rates of transformation and of progression of precursor lesions (de Abreu et al. 2012a). Since the infection is asymptomatic in most cases, it may go by untreated and thus become a persistent infection that favours chronic inflammation. Moreover, we found that only 25% of patients positive for *C. trachomatis* described a symptom, such as abundant vaginal discharge or pruritus, which once again underscores the importance of molecular screening. It is known that *C. trachomatis* induces not only chronic inflammation, but also damage to epithelial tissue and inflammatory pelvic disease (Bellaminutti et al. 2014). Infection occurs in immature endocervical cells, prompting an epithelial transformation called metaplasia. Therefore, metaplasia may be seen as a potential factor associated with high risk of HPV infection, since this virus preferably infects metaplastic epithelia (Urbina et al. 2010, Deluca et al. 2011). The present study shows the association between *C. trachomatis*infection and the presence of characteristic cytological changes such as ASCH-H and different HPV genotypes. In spite of the low number of *C. trachomatis* and HPV co-infections observed (4, 2.4%), an association was noticed both for single and multiple high-risk HPV genotype infections, lending strength to the hypothesis that infection with *C. trachomatis* may be one of the cofactors of CC, together with inflammation and infection with HPV.

It is important to consider that our study has limitations, as the small sample size, especially considering with intraepithelial alterations in cervix samples to confirm the associations observed. Besides, the follow up of these patients is another perspective to evaluate the persistence of the different microorganism.

In light of the clear association between the emergence of lesions and the possibility of progression into cervical cancer, the molecular investigation of HPV and *C. trachomatis* should be considered in prior cytological screening. Such indication should be interpreted in light of the limitations of conventional diagnosis procedures to detect important microorganisms that may lead to inflammation, worsening the carcinogenesis scenario. Therefore, the association of different techniques affords a more sensitive and specific diagnosis, and helps in the early identification and follow-up of precursor lesions of cervical cancer.
